# Electrochemical Behavior of Al/Mg Alloys Immobilized in a Magnesium Potassium Phosphate Cement-Based Mortar

**DOI:** 10.3390/ma16155415

**Published:** 2023-08-02

**Authors:** Gabriel Poras, Céline Cau Dit Coumes, Pascal Antonucci, Céline Cannes, Sylvie Delpech, Stéphane Perrin

**Affiliations:** 1 CEA, DES, ISEC, DPME, SEME, LFCM, Université de Montpellier, 30207 Bagnols-sur-Cèze, France; 2 IJCLab, CNRS/IN2P3, Université Paris-Saclay, 91405 Orsay, France

**Keywords:** radioactive waste conditioning, magnesium potassium phosphate cement, aluminum, magnesium, corrosion, electrochemical impedance spectroscopy, gas chromatography

## Abstract

Portland cement is extensively used for the conditioning of radioactive waste. However, its high alkalinity is a serious obstacle to the stabilization of waste containing aluminum metal since aluminum is oxidized by the pore solution with the production of dihydrogen. This work investigates the potential of an alternative binder, magnesium potassium phosphate (MKP) cement, for the stabilization of Al–Mg alloys comprising 2 to 4.5 wt% of Mg and other metallic impurities. The objective is to assess the influence of the alloy composition on its reactivity in the cementitious matrix at earlier ages, as well as at later ages, when the cement has reached a significant reaction degree. Two complementary techniques are used. Gas chromatography shows that the dihydrogen release, resulting from the corrosion process, is not influenced by the magnesium content in the alloy. Electrochemical impedance spectroscopy provides qualitative information about the corrosion but also makes it possible to assess the corrosion current using an equivalent electrical circuit linked to the kinetic parameters of the postulated corrosion mechanism. Over a one-year period, the corrosion current of the alloys, regardless of their Mg content, is reduced by almost three orders of magnitude in MKP mortar as compared to Portland-cement-based mortar.

## 1. Introduction

Reprocessing of spent fuel designed for natural uranium—graphite—gas reactors has produced some radioactive wastes containing aluminum–magnesium alloys and categorized as low-level or intermediate-level (LL-IL) radioactive wastes [1]. These metal pieces, which were used as cartridges for transportation of spent fuel claddings before their reprocessing, are made of Al/Mg alloys belonging to the 5XXX series according to ANSI H35.1 standard [2]. Their magnesium content is typically comprised between 3 wt% and 5 wt%. Magnesium shows a rather high solubility in aluminum in the solid state (14.9 wt% at 451 °C, 1.7 wt% at room temperature [3]). When present in a solid solution, it significantly improves the corrosion resistance of aluminum by slowing down the rate of the cathodic reaction [4]. Al/Mg alloys of the 5XXX series are thus well suited for structural use in aggressive salty environments [5]. The β-phase (Mg_2_Al_3_) can, however, precipitate in systems containing more than ~3% Mg and weaken the alloy by depleting the Mg fraction in the solid solution [4].

**Figure 1:**
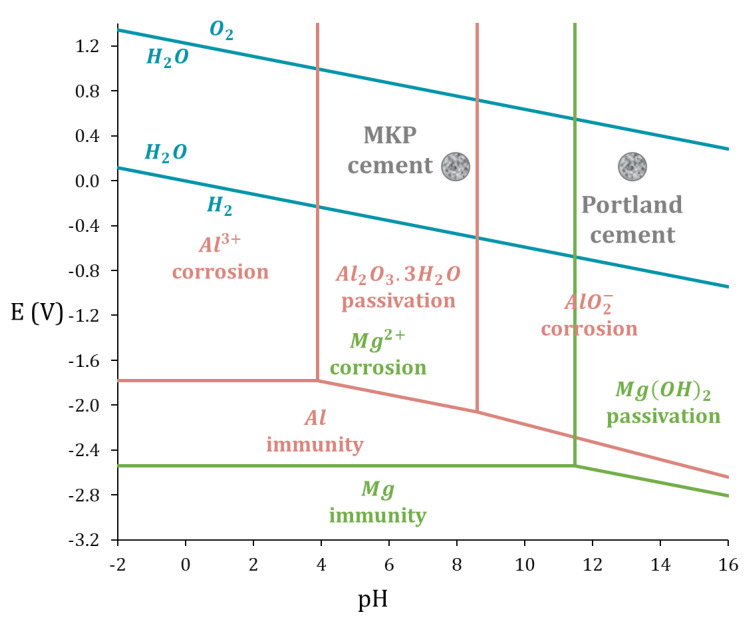
E–pH diagram for pure Al (pink) and pure Mg (green) in water at 25 °C (derived from [10], $[Al^{3+}]=[AlO_{2}^{-}]=[Mg^{2+}]=10^{-6}mol/L$).

Legacy metallic wastes made of Al/Mg alloys need to be stabilized and solidified before their final disposal in a near-surface repository. Portland cement (PC) is extensively used for the conditioning of LL–IL radioactive waste [6,7]. However, its high alkalinity is a serious obstacle to aluminum stabilization, this metal being oxidized by the pore solution of the binder with the production of dihydrogen resulting from reduction of water [8,9]. Its passivation due to the formation of a protective layer of alumina only occurs for pH values within the range of 3–9 [3,10] (Figure 1). Investigations have thus been undertaken in order to find alternative binders showing better chemical compatibility with this metal [8,11,12,13], and promising results have been obtained with magnesium potassium phosphate (MKP) cements [8,13]. These binders have been investigated since the 1990s for the stabilization of radioactive waste [14,15,16,17,18,19,20]. They are prepared by mixing basic magnesium oxide with acidic potassium dihydrogen phosphate (KH_2_PO_4_) in the presence of water, which explains their classification as acid-base binders. They mainly yield K–struvite (MgKPO_4_·6H_2_O), a magnesium potassium phosphate hydrate, following (Equation (1)).

(1) $$MgO+{KH}_{2}{PO}_{4}+5H_{2}O\to {MgKPO}_{4}\cdot {6H}_{2}O$$

The reaction is highly exothermic. To control the setting and keep moderate thermal output, a retarder such as boric acid [21,22], citric acid [23], aluminum nitrate [24], or thiosulfate [25] is commonly used, and the cement is blended with supplementary cementitious materials such as fly ash [26], wollastonite [27], metakaolin, or pumice [28]. MKP cement prepared from equimolar amounts of MgO and KH_2_PO_4_ (Equation (1)) has a chemical water demand corresponding to a w/c ratio of 0.51 (where w stands for the mass of water and c for the mass of MgO + KH_2_PO_4_). With such a ratio, a fluid grout is obtained after mixing. Unlike Portland cement, there is no need to provide excess water beyond the chemical demand to obtain good workability. The residual pore solution volume in MKP binder with advanced reaction is thus generally very low, which is an asset to mitigate metal corrosion. The second advantage results from the composition of the pore solution, with a pH close to 8, which falls within the passivation domain of Al metal [8,29,30]. However, this near-neutral pH is expected to be detrimental to Mg metal passivation, as shown via the E–pH diagram of magnesium (Figure 1) [10]. Studies about the immobilization of Mg–Zr alloy radwaste have shown that binders having initially a highly alkaline pore solution (such as metakaolin and blast-furnace slag activated by concentrated sodium silicate and sodium hydroxide, respectively [31,32,33]), are more appropriate candidates to mitigate the corrosion of magnesium. Data about Al–Mg alloys are still very limited. Perona et al. [30] recently investigated the corrosion of Al/Mg 5754 alloy, comprising 3.5 wt% Mg, in a MKP mortar immersed in deionized water. Thanks to polarization resistance measurements, it was shown that the corrosion of the alloy is comparable to that of pure Al metal over the first two weeks of hardening of the MKP cement. Dihydrogen production was then assessed from the corrosion current density derived from the experimental polarization resistance values and was found to be significantly smaller, by at least one order of magnitude, than in a Portland-cement-based matrix. The objective of our work is to complement these first findings by:

- varying the Mg content in the alloy (from 2.0 to 4.5 wt%) and comparing the results with those achieved for pure Al and Mg metals,
- investigating the corrosion of Al–Mg alloys at later ages since the reaction of the MKP cement continues to progress after 14 d,
- using impedance spectroscopy at the open circuit potential in order to avoid any perturbation of the metal–matrix interface due to polarization effects,
- validating the approach consisting of assessing the dihydrogen production from the corrosion current by comparing it to direct H_2_ measurements using gas chromatography.

## 2. Experimental

### 2.1. Aluminum-Magnesium Alloys

**Figure 2:**
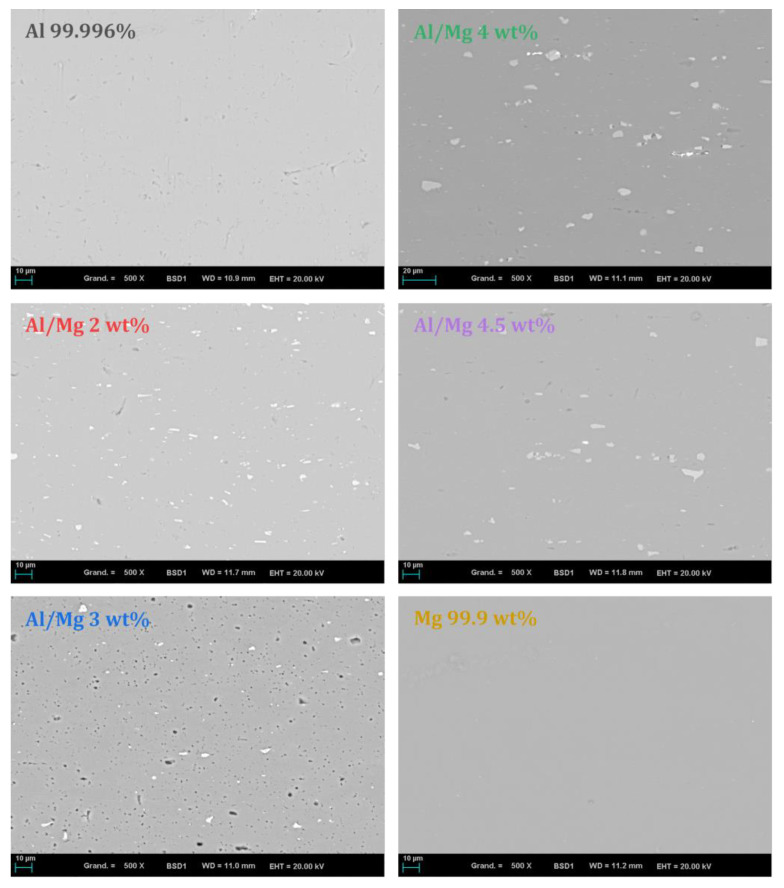
BSE images of metals and Al/Mg alloys studied (polished section, ×500).

To mimic legacy radioactive wastes, different commercial Al/Mg alloys were selected for this study. Their compositions are given in Table 1. Pure aluminum and pure magnesium were compared to mixed alloys of the 5XXX series with a Mg content varying from 2.0 to 4.5 wt%. The chemical composition of the metals and alloys was established using ICP-AES after full dissolution in an acidic solution ($HCl+HNO_{3}+HF$). The composition of the Al/Mg alloys was compliant with the American National Standard ANSI H35.1, the main impurities being iron, manganese, silicon, and chromium. The iron content was slightly higher for Al/Mg2 and Al/Mg4.5 alloys than for the other metals. Metallographic analyses were also performed on polished samples using SEM-EDX. Backscattered electron images are presented in Figure 2. For pure aluminum and pure magnesium, no inclusions were visible. For Al/Mg alloys, inclusions containing mostly iron, but also manganese, were identified (grey/white particles). Dark zones on pure Al and Al/Mg 3 wt% alloy appeared to be small porosities in the matrix. Note that it was impossible to depict the possible presence of silicon in inclusions as the preparation of the samples involved a polishing step using micro-silica.

Metals were supplied as large plates with a thickness reported in Table 1. Al and Al/Mg alloys were cut to the requested dimensions using a wire saw under water cooling. Mg was cut using a wire cutter under dry conditions. Before starting an experiment, the metallic samples were cleaned using isopropanol and immersed in an acidic solution $([H_{2}SO_{4}]=0.1\;mol/L$) for 30 s in order to remove the native oxide layer on their surface. They were then rinsed again with isopropanol, dried at room temperature under air for 5 min, and immediately introduced in the MKP mortar.

### 2.2. MKP Mortar

The mix design of the mortar prepared using MKP cement is given in Table 2. The Mg/P molar ratio and the water-to-cement weight ratio (where cement stands for the mass of MgO + KH_2_PO_4_) were set to 1 and 0.51, respectively, which correspond to the stoichiometry of K-struvite formation following equation (Equation (1)). Boric acid was added as a set retarder with a B(OH)_3_-to-cement weight ratio set to 0.02. Low-CaO fly ash and sand were added with weight ratios of 1 compared to cement. Details on the composition and physical properties of the raw materials are given in the work of Chartier et al. [34] (fly ash: Cordemais 2013). Powders (MgO, KH_2_PO_4_, fly ash, and sand) were premixed in a standardized (EN 196-1 [35]) mortar mixer. Boric acid was dissolved in deionized water and this mixing solution was added to the powders. Mixing was performed for 3 min at low speed. The mortar was then cast in 100 mL-polyethylene cells containing the metal samples as described hereafter.

The pore solution pH of the mortar was measured whenever an electrochemical measurement was performed using ex situ leaching using the protocol described by Alonso et al. [36].

### 2.3. Gas Analysis

Gas chromatography (GC) was used to monitor the production of dihydrogen resulting from aqueous corrosion of metals. Indeed, as aluminum and magnesium are oxidized, water is reduced, which produces dihydrogen, as shown by (Equations (2) and (3)).

(2) Al+3H2O→Al3++32H2+3OH−

(3) $$Mg+2H_{2}O\to {Mg}^{2+}+H_{2}+2OH^{-}$$

**Figure 3:**
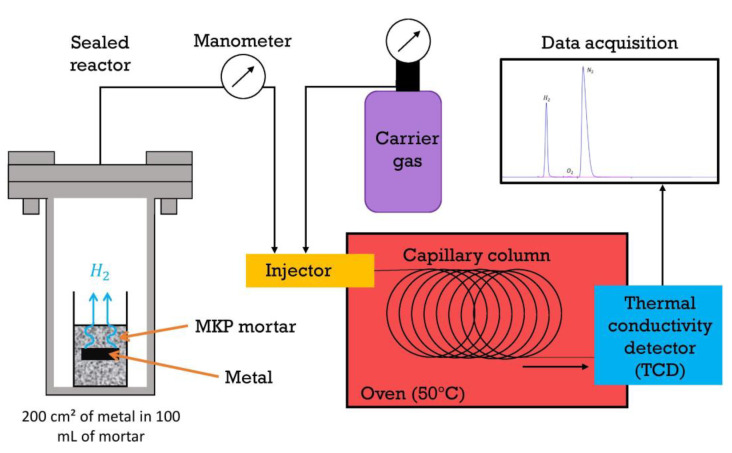
Setup used to monitor the gas released by mortars encapsulating Al, Mg, and Al/Mg alloys via gas chromatography.

Five metal pieces of a given alloy (with the following approximate dimensions: 10 cm long and 2 cm wide) developing a total surface area of c.a. 200 cm^2^ were embedded in 100 mL fresh MKP mortar in a single polyethylene cell. The cell was then introduced in an airtight stainless steel reactor, as shown in Figure 3. The reactor was placed under a nitrogen atmosphere by applying a vacuum (until 150 mbar), and filling with nitrogen back to 750 mbar, this step being triplicated. The reactor was stored at ambient temperature (22 ± 2 °C). Gas aliquots were sampled after periods of time ranging from 1 day to 1 year for H_2_ analysis by gas chromatography (Varian 3400, argon carrier gas, capillary column, thermal conductivity detector, detection limit corresponding to 0.01% dihydrogen in the headspace of the reactor, single-point routine calibration curve using a reference sample containing 0.5% dihydrogen, and periodic calibration with five standards from 0 to 5% H_2_). The loss of H_2_ at each sampling was taken into account to calculate the total volume of H_2_ released by the cemented metal (see Appendix A). This latter ($V_{H_{2}}$, expressed in liters), was standardized with respect to the initial surface area S of metal in contact with the mortar ($V_{H_{2}/S}=V_{H_{2}}/S$). Uncertainties on the reactor pressure (P) and volume (V), as well as on the dihydrogen content and metal surface area, were taken into account to assess the experimental error (see calculation in Appendix A).

### 2.4. Potentiometric and Electrochemical Impedance Spectroscopy Measurements

**Figure 4:**
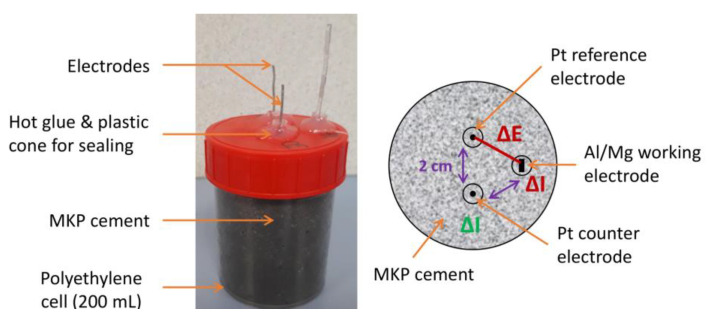
Electrochemical setup for Al, Mg, and Al/Mg alloys embedded in MKP mortar: experimental cell (**left**) and position of the electrodes (**right**).

For the electrochemical experiments, a specific cell was designed (Figure 4), comprising three electrodes (1 in Al, Mg, or Al/Mg alloy and 2 in Pt, 1 used as pseudo-reference and one as counter electrode) embedded in the MKP mortar. Commercial reference electrodes such as the Ag/AgCl electrode were not used for two reasons: their glass frit would be clogged by the cement matrix, and ions from the electrolyte (especially chlorides) would diffuse in the pore solution, thus modifying its composition. Solid electrodes such as the MnO_2_/Mn reference electrode are widely used in concrete for rebar corrosion studies [37]; however, their high alkalinity precludes their use in near-neutral MKP cement-based materials. The platinum wire (purity 99.5%, 1 mm diameter) was supplied by ThermoFisher (Waltham, MA, USA). They were cleaned with the flame of a gas burner and rinsed with isopropanol before use. The Al and Al/Mg alloy electrodes were platelets 10 cm long and 0.3 cm wide, which were cut and cleaned as described in Section 2.1. The Mg electrode was a magnesium wire (purity 99.9%, 1 mm in diameter) supplied by Goodfellow. Three holes were drilled in the lid of the polyethylene cell. Electrodes were fixed using plastic cones and glue to obtain an exact 4 cm length of metal plunged in the mortar and an approximate distance of 2 cm between the pseudo-reference, the working, and the counter electrodes. The surface areas of the working electrodes ranged between 3 and 3.5 cm^2^ (values given for each sample in Appendix C), and that of the counter electrode was close to 1.3 cm², thus giving an area ratio (working/counter electrode) comprised between 2.3 and 2.8. 

The cell was filled with 100 mL of fresh MKP mortar. The lid with the electrodes was put in place and tightly sealed.

Potentiometric and electrochemical impedance spectroscopy (EIS) measurements were regularly performed on mortars aged from 1 day to 1 year using a potentiostat (model PARSTAT 4000+, AMETEK, Berwyn, PA, USA) piloted with VersaStudio^®^ software (version n°2.62.2). The open circuit potential (OCP) was first measured. The impedance spectrum of the system was then acquired at OCP by applying a small sinusoidal potential disturbance Δ*E*(*f*) of 10 mV between the Al, Mg, or Al/Mg alloy working electrode and the pseudo-reference electrode, with a frequency varying from 2 × 10^6^ Hz to 0.1 Hz (10 points per decade). The resulting current variation Δ*I*(*f*), going through the working and counter electrodes, was measured to determine the impedance of the system defined by (Equation (4)).

(4) $$Z\left(f\right)=∆E\left(f\right)/∆I\left(f\right)\;$$

Between the two measurements, the samples were cured at room temperature. The tightly closed cells prevented, as far as possible, any desiccation of the mortar. Experiments (preparation of electrochemical cell and EIS measurements) were triplicated for each metal and showed good repeatability.

The impedance spectra were fitted using a homemade Python™ (version 3.9.12) fitting script and ZView^®^ software (version 3.5g) [38]. The experimental error on the faradaic resistance was estimated during the fit by assessing the range of values for which the fit was considered to be accurate. 

The polarization resistance measurement method is widely used for corrosion studies in cementitious matrices [29,39]. EIS was preferred in this work, however, since it made it possible to investigate both the electrochemical behavior of the matrix and the corrosion process.

## 3. Results

### 3.1. Dihydrogen Release Measured Using Gas Chromatography

**Figure 5:**
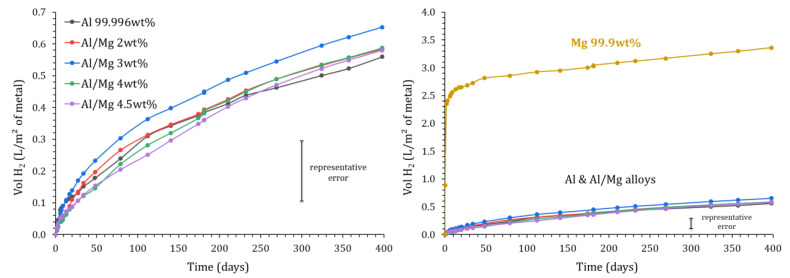
Variation of the normalized H_2_ production measured by gas chromatography for Mg, Al, and Al/Mg alloys as a function of immersion time in MKP mortar (P = 1 atm, T = 25 °C).

Figure 5 shows the H_2_ release measured using gas chromatography in the different samples over time. As expected from the potential—pH diagrams (Figure 1), the cumulative volume of gas obtained for pure magnesium increases quickly to reach 2.5 L/m^2^ after one week, which is almost 10 times higher than for pure aluminum and Al/Mg alloys. However, after one week, the dihydrogen production slows down and reaches a plateau. The H_2_ amount released after 400 d corresponds to the corrosion of only 0.2 mol% of the magnesium present in the mortar sample, meaning that the plateau does not result from Mg metal exhaustion but rather from two possible processes:

(i) water depletion via the cement reaction and the corrosion processes, and
(ii) precipitation of a protective layer of a phosphate phase such as K-struvite (MgKPO_4_·6H_2_O).

The cumulative volume of gas released ranges between 0.56 and 0.65 L/m^2^ after 400 d for pure Al and the different Al/Mg alloys. Note that the experimental error is important, for instance, close to ±0.10 L/m^2^ for Al/Mg3 alloy at 400 d. At a given characterization time, the variations observed for the different alloys are non-significant. Consequently, the GC-measured H_2_ release does not seem to be notably influenced by the Al alloy grade. Further, the gas release when pure aluminum is encapsulated in a Portland cement paste reaches 40 L/m^2^ in only 4 h [8]. These results thus confirm that MKP cement seems more adapted than Portland cement for the conditioning of pure Al, but also of Al alloys containing small amounts of magnesium.

### 3.2. Potentiometric and Electrochemical Impedance Spectroscopy Measurements on Al, Mg, and Al/Mg Alloys in MKP Mortar

#### 3.2.1. Evolution of the Open Circuit Potential with Ongoing Cement Reaction

**Figure 6:**
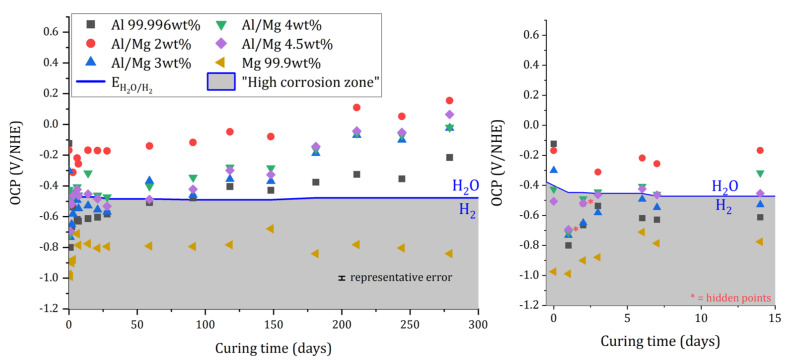
Variation of Open Circuit Potential (OCP) measured on Al, Mgm, and different Al/Mg alloy electrodes vs. normal hydrogen electrodes as a function of immersion time in MKP.

The open circuit potential (OCP) of pure Al, pure Mg, and the different Al/Mg alloy electrodes embedded in the MKP mortar was recorded as a function of time versus a platinum quasi-reference electrode embedded in the MKP mortar. The relationship established by Bouhier et al. [40] and giving the potential of Pt wire versus NHE as a function of pH was then used to calculate the OCP values against a normal hydrogen electrode (NHE). The pH varied from 7.6 at 1 d to 8.0 at 7 d and then remained almost constant. In Figure 6, the OCP values are compared to the potential of the H_2_O/H_2_ redox couple ($E_{H_{2}O/H_{2}}$) corresponding to water reduction, and calculated using the Nernst equation for pH values relevant to MKP mortar pore solution. Therefore, the high aqueous corrosion zone in MKP mortar corresponds to OCP values below $E_{H_{2}O/H_{2}}$ (grey zone in Figure 6), i.e., below ≈−0.48V/NHE.

Whatever the investigated metal, the main evolution of OCP is observed during the first days of immersion. Initially, all the potentials are lower than the water reduction potential, indicating the formation of H_2_, which is consistent with the GC measurements. Then, OCP increases quickly. In the case of magnesium, it tends to have a constant value, close to −0.8 V/ENH but still below $E_{H_{2}O/H_{2}}$, and thus corresponds to a corrosion potential, as expected [10]. However, the large difference between the Mg standard potential (−2.5 V/ENH) and the corrosion potential indicates some passivation of the electrode, likely due to the precipitation of a magnesium phosphate phase at the interface. This agrees well with the decrease in the H_2_ release observed experimentally in Section 3.1 (Figure 5). 

The OCP values of Al and Al/Mg alloys exceed the reduction potential of water after some time. In this case, the formation of a protective layer at the metal/matrix interface leads to the behavior of an “inert” electrode. The OCP values cannot be considered as corrosion potentials anymore but rather as equilibrium potentials controlled by redox systems, which may differ for Al and Al–Mg alloys given the gap in the recorded data (up to c.a. 400 mV between the different metals). 

#### 3.2.2. Qualitative Evolution of Impedance Spectra

**Figure 7:**
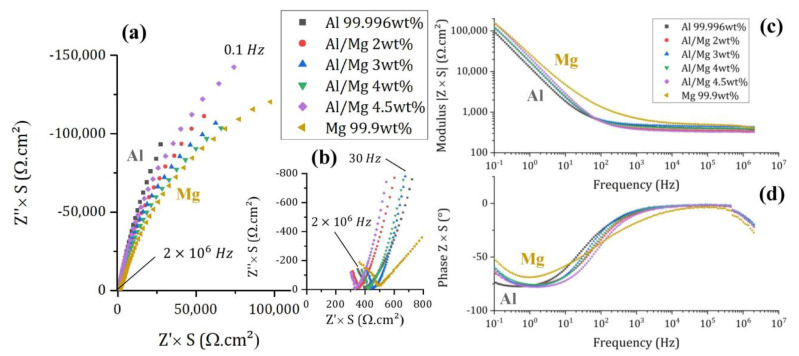
Electrochemical impedance spectra of Al, Mg, and Al/Mg alloys embedded in MKP mortar after 6 days of curing at 20 °C, with an amplitude of 10 mV. (**a**) Nyquist diagrams—(**b**) Zoom of the Nyquist diagrams at high frequencies—(**c**,**d**) Bode modulus and phase diagrams.

Impedance spectra acquired on pure Al, Mg, and the different Al/Mg alloys embedded in MKP mortar after 6 d of curing are shown in Figure 7. Since impedance Z is inversely proportional to the electrode surface area S [29], normalized impedance Z×S is plotted on Nyquist and Bode diagrams in order to compare data obtained with electrodes having different surface areas.

The small impedance values, measured at high frequencies (Figure 7b), mainly result from the mortar contribution. On the contrary, the high impedance values measured at low frequencies are linked to the corrosion process. The Nyquist diagrams at low frequencies can be qualitatively analyzed as follows: the more vertical the plot, the slower the corrosion rate. Therefore, after 6 d of curing, corrosion of Al is slower than that of Mg in MKP mortar, as expected from the E–pH diagrams, and consistently with OCP values. Al/Mg alloys have corrosion rates intermediate between those of Al and Mg when considering slopes on EIS spectra, which suggests that the passivating layer on Al–Mg alloys is less protective than that on pure Al.

The spectrum recorded for pure magnesium at high frequency is quite different, with a slope on the Nyquist diagram close to 45° and higher modulus values. This shape is also observed on the spectrum (not shown here) recorded with an inert Pt working electrode embedded in a mortar sample containing magnesium metal, meaning that it is likely due to a change in the properties of the MKP mortar, possibly due to the strong gas release resulting from Mg corrosion and modifying the pore network.

#### 3.2.3. Quantitative Analysis of Electrochemical Impedance Spectra

- Corrosion mechanism and equivalent electrical circuit

In order to investigate more thoroughly the corrosion mechanism of Al metal and Al alloys, a quantitative analysis based on EIS was performed. 

**Figure 8:**
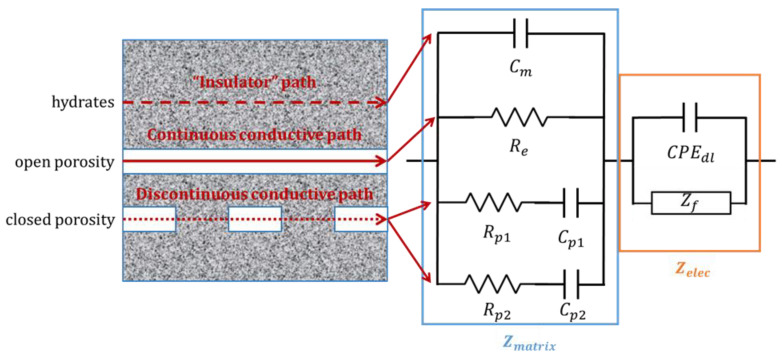
Conductive paths through the microstructure of concrete (**left**), as described by Song et al. [41]; schematic representation and equivalent electrical circuit used in this study (**right**).

To model the impedance spectra, it is necessary to take into account the cement matrix contribution to the impedance and the faradic impedance linked to the corrosion mechanism. Song et al. (2000) [41] have described the cement mortar contribution to the impedance by distinguishing different conductive paths (Figure 8):

 - the “insulator” conductive path (ICP) through the hydrates is modeled using a pure capacitance C_m_,
 - the continuous conductive path (CCP) through the open porosity and the electrolyte is modeled using a pure resistance R_e_,
 - discontinuous conductive paths (DCP) through the closed porosity of the matrix are modeled using a resistance and a capacitance in series.

In MKP mortar, two DCPs in parallel ($R_{p1}-C_{p1}//R_{p2}-C_{p2}$) are required to fit the experimental data, which was attributed to the progressive refinement of the pore network with ongoing cement reaction [42]. Pure capacitance and constant phase angle element (CPE) have been considered in the literature to describe the contribution of the bulk material to the impedance [43]. CPE is preferentially used for complicated materials and makes it possible to account for a deviation from pure capacitance (Equation (5)).

(5) $$Z_{CPE}=\frac{1}{C_{CPE}*{\left(j\omega \right)}^{\beta }}$$

Concerning MKP mortar, previous work [29] has shown that parameter β remains close to 1, meaning that, as an initial approach, a pure capacitance can be used to model the bulk material.

As the metallic electrodes are immersed in MKP mortar, an electrical double layer of ions forms at the interface between the conductive electrodes and the electrolyte. This phenomenon is represented by a constant phase element ($CPE_{dl}$), in parallel with the faradaic impedance accounting for corrosion. As already reported in the literature [42], it is necessary to consider a CPE instead of a pure capacitance to describe the electrical double layer, probably because of the roughness of the solid-electrode interface.

An electrochemical mechanism in four steps, already presented in Delpech et al. [29], is used to describe the Al corrosion process. For Al/Mg alloys, two steps are added to the postulated mechanism.

(6) $$Al\;oxidation:Al+s_{Al}\overset{{k_{ox}}_{Al}}{\to }Al^{+III},s_{Al}+3e^{-}$$

(7) $$Mg\;oxidation:Mg+s_{Mg}\overset{{k_{ox}}_{Mg}}{\to }{Mg}^{+II},s_{Mg}+2e^{-}$$

(8) Water reduction:H2Oelectrode+e−→kred12H2+OH−

(9) $$Water\;diffusion:{H_{2}O}_{matrix}\overset{D}{\to }{H_{2}O}_{electrode}$$

(10) $$Al^{+III}\;desorption:Al^{+III},s_{Al}\overset{K_{1}}{\to }Al^{+III}+s_{Al}$$

(11) $${Mg}^{+II}\;desorption:{Mg}^{+II},s_{Mg}\overset{K_{2}}{\to }{Mg}^{+II}+s_{Mg}$$

The mechanism takes into account the oxidation of aluminum and magnesium (Equations (6) and (7)), the reduction in water at the electrode (Equation (8)), water diffusion from the matrix to the electrode (Equation (9)), and desorption of $Al^{+III}$ and $Mg^{+II}$ from electroactive sites on the electrode surface (Equations (10) and (11)). This latter process can account for the diffusion of ions from the electrode to the matrix but also for passivation via precipitation of Al- or Mg-containing minerals on the electrode. Specific electroactive sites are assumed for Al and Mg species.

**Figure 9:**
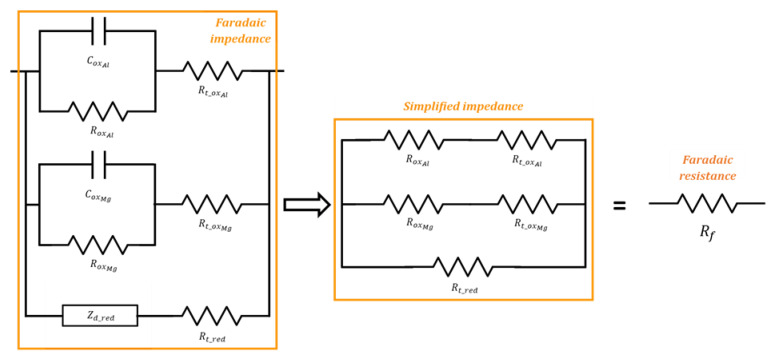
Equivalent electrical circuit to simulate the electrochemical impedance diagrams recorded on Al/Mg electrodes (**left**); simplified circuit equivalent to a pure faradaic resistance (**right**).

An equivalent electrical circuit is then derived from this mechanism using Fick’s law and the Butler–Volmer equation following the method described in [29]. The equivalent circuit comprises three branches in parallel (Figure 9). The first one, corresponding to Al oxidation, is made of a charge transfer resistance $R_{t_{ox}}$ and a $R_{ox}//C_{ox}$ circuit representing the contribution of the desorption/passivation step. The second branch is similar but accounts for Mg oxidation and desorption/passivation. The third one, accounting for reduction, also includes a charge transfer resistance in series with a Warburg impedance of diffusion. All the electrical parameters in Figure 9 are then expressed as a function of the kinetic parameters of the Al/Mg corrosion mechanism. The electrical parameters are adjusted by fitting the impedance spectra recorded after increasing periods of curing. Preliminary fitting results show that the faradaic impedance can be simplified for Al/Mg alloys by neglecting the $Z_{d\_red}$ and $C_{ox}$ elements. Indeed, the impedance spectra of these metals (pure Al and Al/Mg alloys) do not show any contribution of water diffusion (which would produce a straight line with a slope at 45° on the Nyquist diagram at low frequencies), and the diffusion parameters can be varied over a large domain without any influence on the modeled spectra. Secondly, the $C_{ox}$ capacitances are very low both for Al and Mg branches due to passivation of the electrode ($C_{ox}\sim 10^{-13}F$), and are thus negligible compared to the double-layer capacitance $CPE_{dc}$ ($C_{dc}\sim 10^{-5}F$). Consequently, the faradaic impedance can be simplified by considering five resistances only, which, finally, are equivalent to a pure faradaic resistance $R_{f}$ (Figure 9).

**Figure 10:**
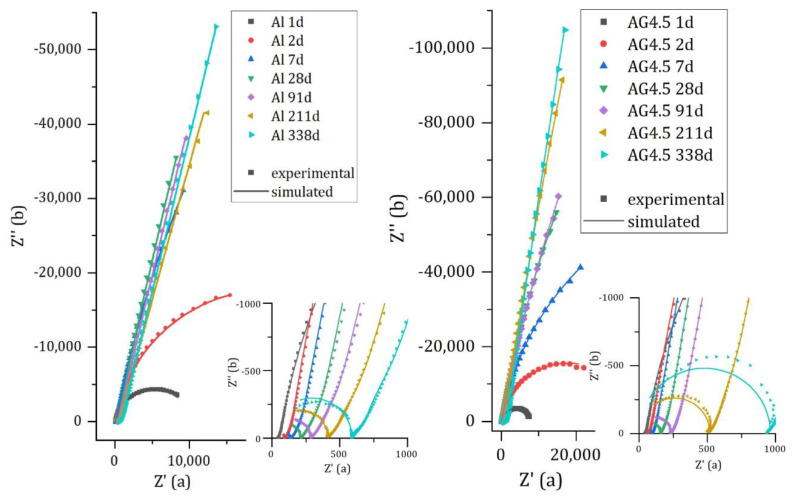
Experimental and simulated impedance spectra of pure Al (**left**) and Al/Mg 4.5 wt% alloy (**right**) embedded in MKP mortar after increasing curing times at 20 °C.

**Figure A1:**
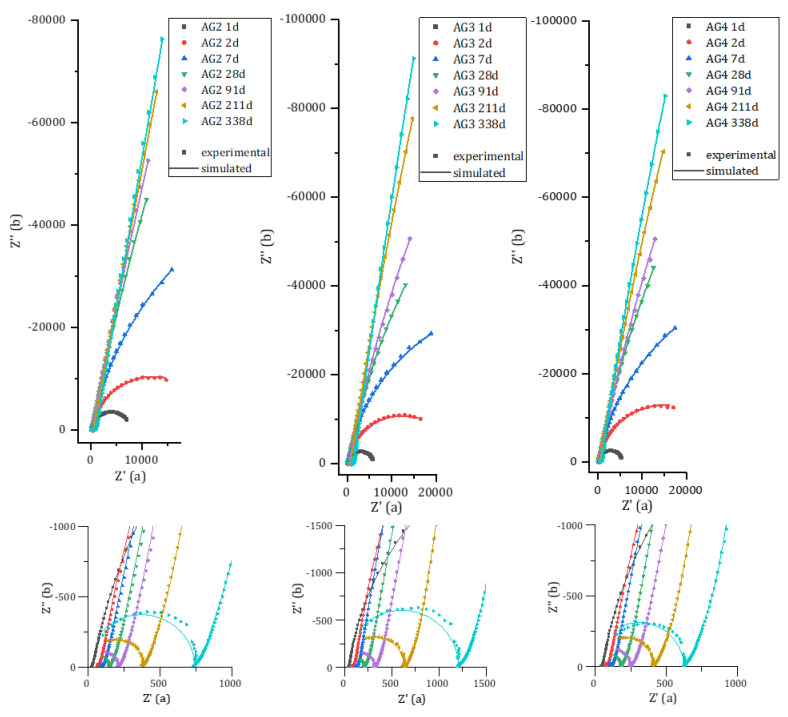
Experimental and simulated impedance spectra of Al/Mg 2 wt%, 3 wt%, and 4 wt% alloys embedded in MKP mortar after increasing curing times at 20 °C.

Figure 10 compares the experimental and calculated spectra for pure aluminum and Al/Mg4.5 alloy encapsulated in MKP mortar. The spectra for Al/Mg2, 3, and 4 alloys are given in Appendix B (Figure A1). The simulated spectra fit the experimental data rather well, except at high frequencies and long time (more than 200 d), where the use of a pure capacitance $C_{m}$ for the matrix does not enable to model accurately the matrix contribution. The optimized electrical parameters for pure Al and all Al/Mg alloys studied are given in Appendix C (Table A1, Table A2, Table A3, Table A4 and Table A5). Two main changes occur over time on the Nyquist diagrams. Firstly, at high frequencies (zoom in Figure 10—right), the mortar contribution to the impedance evolves during cement reaction, with an increase in the mortar electrical resistance. Secondly, the impedance at low frequencies increases over time, and the shape of the Nyquist diagram becomes almost vertical, meaning that corrosion strongly slows down.

- Evolution of the mortar contribution to the impedance

**Figure 11:**
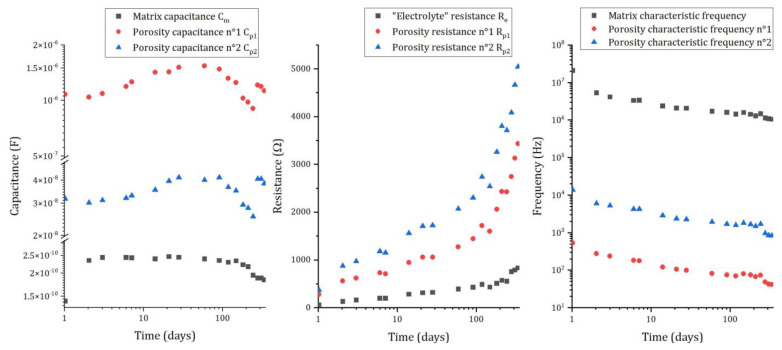
Evolution of the electrical parameters representing the matrix contribution to the impedance for pure aluminum embedded in MKP mortar (surface of electrode S = 2.94 cm²): capacitances (**left**), resistances (**middle**), and characteristic frequencies (**right**).

The mortar matrix has an important contribution to the impedance of the system, which evolves as the reaction of cement progresses. Figure 11 shows the evolution of the electrical parameters relative to the matrix for pure aluminum embedded in MKP mortar. 

As explained in the previous section, three capacitances and three resistances are used to model the matrix contribution to the impedance. The capacitances (Figure 11 left) of the different branches differ by almost two orders of magnitude ($C_{m}\sim 2\times 10^{-10}F\ll C_{p2}\sim 3\times 10^{-8}F\ll C_{p1}\sim 1.5\times 10^{-6}F$), but show almost no change with ongoing reaction. Inversely, the resistances (Figure 11—center) strongly increase with time (for instance, from 54 Ω after 1d to 831 Ω after 338 d for the electrolyte resistance). This increase results from the progress of the cement reaction, which consumes water and refines the porosity via precipitation of cement hydrates [44,45,46]. Further, the pore solution, which is strongly oversaturated with respect to K-struvite in the first stages of the reaction, evolves towards thermodynamic equilibrium at later ages. Thus, three processes can contribute to an increase in the following resistances:

 (i) progressive desaturation of the pore network due to water consumption,
 (ii) decrease in the mobility of dissolved species in the residual pore solution due to depercolation of the pore network, and
 (iii) decrease in their concentrations due to progressive equilibration.

Discontinuous conductive paths through the closed porosity of the matrix are represented electrically via a resistance and a capacitance in series, which can be considered in a first approach as high-pass filters passing signals with frequencies higher than the characteristic frequencies [47]. These frequencies are calculated for the different paths (Equations (12)–(14)):

(12) $$f_{c/Matrix}=\frac{1}{2\pi *R_{e}*C_{m}}$$

(13) $$f_{c/Porosityn{}^{\circ}1}=\frac{1}{2\pi *R_{p1}*C_{p1}}$$

(14) $$f_{c/Porosityn{}^{\circ}2}=\frac{1}{2\pi *R_{p2}*C_{p2}}$$

They tend to decrease with time (Figure 11—right), which results from the increase in the resistances. Each branch of the electrical circuit has very different characteristic frequencies, meaning that their contribution to the impedance is limited to specific and restricted domains of frequencies: very high frequencies ($10^{6}-10^{7}$ Hz) for matrix parameters ($R_{e}$ and $C_{m}$), and intermediate frequencies ($10^{2}-10^{4}$ Hz) for closed porosity parameters. Consequently, at low frequencies (below $10^{1}$ Hz), the matrix contribution to the impedance becomes negligible, and the EIS spectra mainly provide information on the faradaic resistance. Since the characteristic frequencies of the cement matrix tend to decrease over time, measurements at very low frequencies (10^−1^ Hz) are mandatory to assess the faradaic part of the impedance.

- Calculation of the corrosion current

The corrosion reaction can be evaluated using the parameter $R_{f}$: the smaller $R_{f}$ is, the more the metal or the alloy is corroded. The corrosion current ($I_{corr}$) is calculated from this fitted faradaic resistance value $R_{f}$. The measurements are performed at the corrosion potential $E_{corr}$; consequently, $I_{corr}$ is equal to the opposite of the reduction current. The latter is expressed using the water reduction equation and assuming negligible water diffusion. Eventually, the corrosion current is given by Equation (15):

(15) $$I_{corr}/S=\frac{RT}{\alpha_{red}F\times R_{f}\times S}(A/m²)$$

with R the molar gas constant (in J.mol^−1^.K^−1^), T the temperature (in K), F the Faraday constant (in C.mol^−1^), S the electrode surface area (in m²), and $\alpha_{red}$ the charge transfer coefficient for water reduction. The charge transfer coefficient $\alpha_{red}$ was determined by Bouhier et al. [42] by fitting I=f(E) stationary curves on Pt electrode in water. A value of $\alpha_{red}=0.5$ was obtained.

This expression is identical to that reported by Bouhier et al. [42] in the case of beryllium corrosion and similar to that established by Delpech et al. for pure Al corrosion [29]. It is also applicable in the case of Al/Mg alloys as it is derived from the water reduction equation (Equation (8) from the corrosion mechanism). Note that the calculated corrosion current is proportional to the surface area of the electrode. Since the surface area of the electrodes used in this study can vary slightly, the corrosion current is normalized by considering $I_{corr}/S$.

**Figure 12:**
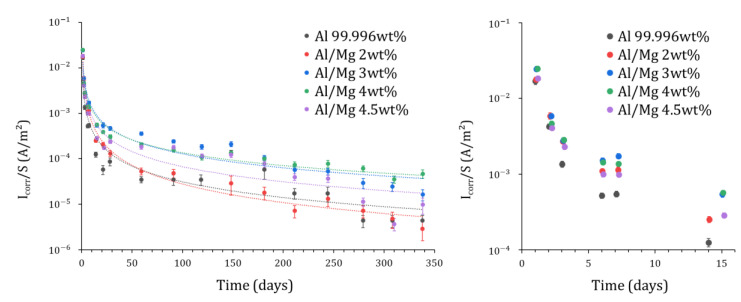
Evolution of the corrosion current $I_{corr}/S$ derived from fitting the EIS spectra for Al and Al/Mg alloys embedded in MKP mortar, normalized by the surface of electrode S.

Quantitative analysis of Mg EIS spectra has been performed but is not shown in this work. The number of spectra available (1 per day) is indeed too small to assess accurately the strong production of H_2_, which mainly occurs at earlier ages, during the first two days after mixing. Figure 12 compares the evolution of the corrosion current obtained for the different aluminum grades. In all cases, the corrosion current decreases quickly during the first month of reaction by at least two orders of magnitude (from $\sim 2\times 10^{-2}A/m²$ at 1 d to $\sim 5\times 10^{-4}A/m²$ or less at 28 d). This drop may result from metal passivation but also from the lack of water available for the corrosion process in the MKP mortar since water is progressively depleted via cement reaction, as mentioned previously. After 340 d, the corrosion current is below 50 µA/m² for all alloys, indicating very slow corrosion. Note that the uncertainty associated with such low current values is rather high (close to 30%). Comparatively, the corrosion current of pure aluminum encapsulated in a Portland cement paste decreases to $\sim 10^{-1}A/m²$ after 200 d [29], and thus exceeds by three orders of magnitude the value measured for pure aluminum in MKP mortar ($\sim 2\times 10^{-5}A/m²)$ (Figure 12). This result confirms again the interest of MKP mortar for the conditioning of Al metal.

The corrosion current can not be simply correlated to the Mg content of the alloy. At earlier ages, all alloys yield close corrosion currents, whereas, at later ages, differences by more than one order of magnitude are observed. At 340 d, for instance, the smallest and highest corrosion current values are recorded for Al/Mg2 ($\sim 3\times 10^{-6}\;A/m²)$ and Al/Mg4 ($\sim 5\times 10^{-5}\;A/m²$), respectively. The differences observed from one alloy to another are too high to be simply explained by uncertainties (≈ 30%) on the corrosion current determination. 

The corrosion currents measured at earlier ages in this work are about 10 times lower than those reported by Perona et al. [30] from polarization resistance measurements ($I_{corr}\sim {1\times 10}^{-2}A/m²$ and $\sim {2\times 10}^{-2}A/m²$ for Al and Al/Mg3 after 10 d, respectively). The different curing conditions of the mortar samples, in tightly closed cells in this work vs. in demineralized water in [30], likely contribute to this discrepancy. In the latter case, the pore network of the mortar remains saturated by water, which likely accelerates metal corrosion and leads to higher corrosion currents.

- Dihydrogen production

Finally, the equivalent H_2_ volume produced per metal surface unit ($V_{H_{2}/S}$) is calculated from the corrosion current using the water reduction equation, Coulomb’s law, and the ideal gas law (Equation (16)):

(16) $$V_{H_{2}/S}\left(t\right)=\frac{1}{S}\times \frac{RT}{P}\times \frac{1}{2F}\int_{0}^{t}I_{corr}dt$$

with R the molar gas constant (in J.mol^−1^.K^−1^), F the Faraday constant (in C.mol^−1^), T the temperature (in K), P the pressure (in Pa), and S the surface area (in m^2^) of the electrode embedded in mortar.

**Figure 13:**
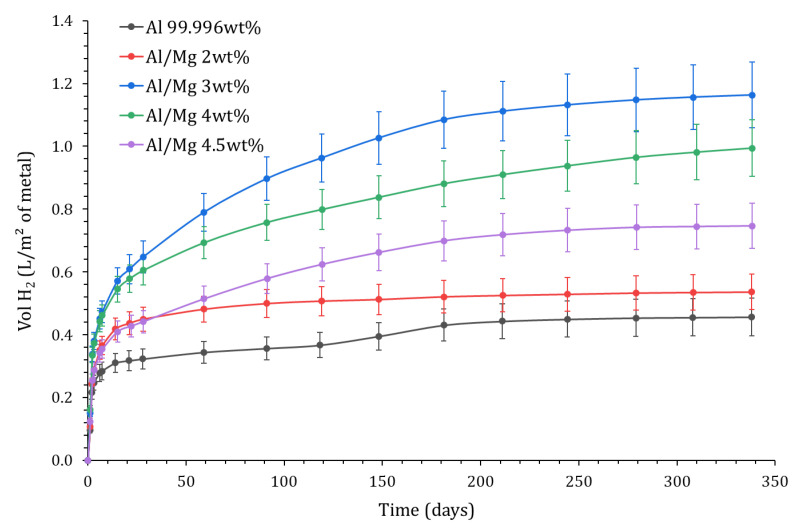
Variation with time of the normalized H_2_ production calculated by EIS as a function of the alloy grade (P = 1 atm, T = 25 °C).

The results are shown in Figure 13 for Al and its alloys. At 340 d, the cumulative volume of H_2_ ranges between 0.5 L.m^−2^ and 1.2 L.m^−2^, and the metals can be sorted in ascending order of H_2_ production: pureAl~Al/Mg2<Al/Mg4.5<Al/Mg4~Al/Mg3. Again, there is no simple correlation between the Mg content of the alloy and the H_2_ release.

## 4. Discussion

### 4.1. Comparison of GC-Measured and EIS-Calculated H_2_ Releases

**Figure 14:**
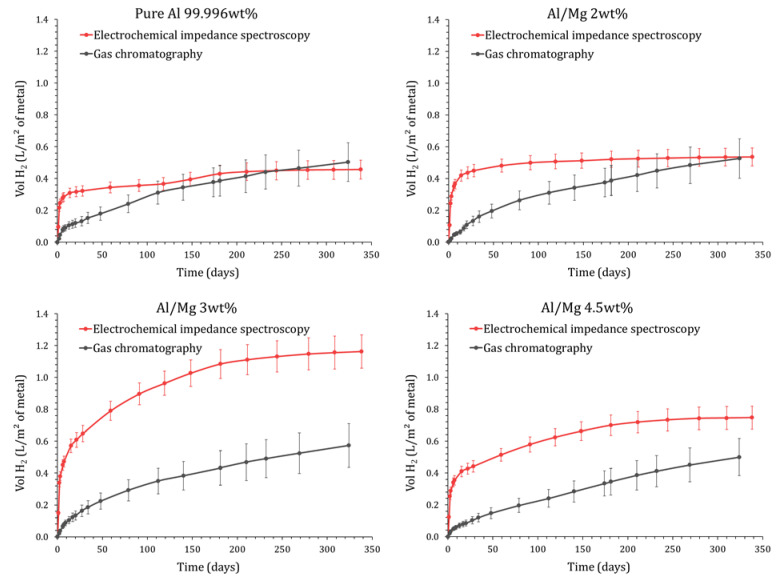
Comparison of the equivalent dihydrogen release by the surface area of metal obtained by gas chromatography and EIS (P = 1 atm, T = 25 °C), for pure Al (**up left**), Al/Mg 2 wt% alloy (**up right**), Al/Mg 3 wt% alloy (**down left**) and Al/Mg 4.5 wt% alloy (**down right**).

The production of dihydrogen derived from electrochemical measurements can be compared to the gas release measured by gas chromatography (Figure 14). Results are presented for pure Al and Al/Mg 2 wt%, 3 wt%, and 4.5 wt% alloys. The behavior of Al/Mg 4 wt% alloy is very similar to that of Al/Mg 4.5 wt%.

For pure aluminum and Al/Mg 2 wt% alloy, the H_2_ production derived from EIS exceeds that measured by GC at earlier ages, but the difference between the two techniques tends to decrease at later ages and, after several months, the two sets of data lead to consistent gas amounts, close to 0.5 L/m². The initial discrepancy between GC and EIS results can result from two factors.

- A fraction of the dihydrogen produced by corrosion may be confined to the porosity of the mortar. Consequently, the H_2_ released in the headspace of the reactor (measured by GC) would only represent a fraction of the H_2_ produced by corrosion (assessed by EIS). With time, this trapped gas may diffuse through the mortar, explaining the better agreement between GC and EIS results. Note that the diffusion coefficient of dihydrogen through cementitious materials strongly depends on the water saturation degree of their pore network. Frizon et al. [48] have shown, for instance, that the diffusion coefficient of H_2_ through a Portland cement paste varies from $\sim 10^{-13}m².s^{-1}$ close to 100% water saturation to $\sim 10^{-6}\;m².s^{-1}$ for water saturation degrees below 60%. In this work, the water saturation degree of the MKP mortar progressively decreases with time, thus making gas diffusion easier as the cement reaction progresses. Note that the desiccation of the sample due to water evaporation in the headspace of the reactor is negligible in both EIS and GC experiments: it would represent a maximum of 23 µL of free water (estimated from Clausius–Clapeyron equation), whereas several milliliters of free water are available in the porosity of the matrix even after a long period of curing (measured by TGA analysis not presented here).
- EIS is a punctual method. The corrosion current is measured at given times and is then integrated to calculate the cumulative H_2_ release, whereas GC directly measures the cumulated gas produced by corrosion. Given the concave-up nature of the corrosion current curve, the trapezoidal rule used for integration very slightly overestimates the value of the integral and, thus, the dihydrogen production derived from EIS.

Nevertheless, the final agreement between the two sets of data tends to validate the postulated corrosion mechanism and the modeling approach of EIS spectra developed in this study.

Regarding the Al/Mg 4.5 wt% alloy, the H_2_ production calculated from EIS always exceeds the GC measurements; this phenomenon is even enhanced for the Al/Mg 3 wt% alloy. Again, the delay due to gas diffusion through the porous matrix may be postulated to explain the smaller gas amounts measured by GC during the first stages of the MKP cement reaction. Nevertheless, at 340 d, the H_2_ production calculated from EIS for Al/Mg 3 wt% alloy ($\sim 1.2\;L/m^{2}$) is still twice higher than the H_2_ release measured by GC ($\sim 0.6\;L/m^{2}$), which can not be simply explained by experimental errors. A second hypothesis is thus considered. The calculation of the equivalent H_2_ production from the corrosion current (see Equation (16)) assumes that aluminum and magnesium are only oxidized by water, with concomitant formation of dihydrogen. However, other oxidation routes may occur without any dihydrogen production. Dissolved oxygen, present in the pore solution of the mortar, may be involved, but its reduction should occur in all experiments, regardless of the metal or alloy embedded in the mortar. Impurities present in the alloys, such as iron, may also play a key role. Iron is very slightly soluble in aluminum and precipitates as intermetallic Al_3_Fe or Al_3_FeMn when the alloy also contains manganese. Such inclusions are present in the Al/Mg alloys under investigation (see Section 2.1). These intermetallics are cathodic with respect to the Al matrix and constitute preferential sites for oxygen reduction. This latter produces local alkalinization [49], which causes an attack of the matrix around the intermetallic particles and explains their detrimental effect on the pitting corrosion of aluminum [50]. Inversely, manganese has a beneficial effect on the pitting corrosion resistance of aluminum [51] by reducing the gap in potential between the matrix and the intermetallics. Silicon plays the same role as manganese with respect to iron, with the resistance to pitting corrosion increasing with the Si/Fe ratio [52]. Nevertheless, in the present study, we failed to establish any simple correlation between the corrosion current of the alloys and their impurity content given in Table 2, suggesting that the microstructure of the alloys also likely plays a role in their corrosion performance and would deserve a more thorough study.

### 4.2. Origin of the Decrease in the H_2_ Release with Time

Both GC and EIS experiments show that the cumulative release of H_2_ levels off after one year. Two hypotheses were considered in Section 3.1 to explain such evolution: *(i)* lack of water, which is consumed by the cement reaction and corrosion process, or *(ii)* precipitation of a protective layer on the surface of the metal. In order to discriminate between these hypotheses, resaturation experiments were conducted on the samples devoted to GC experiments.

**Figure 15:**
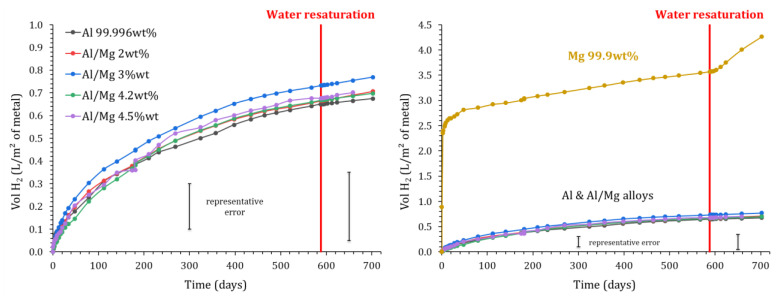
Influence of MKP mortar resaturation using water on the normalized H_2_ production (measured by gas chromatography) due to Mg, Al, and Al/Mg corrosion (P = 1 atm, T = 25 °C).

After 590 days of evolution, the MKP mortars encapsulating metals were removed from the reactors connected to GC. The samples were placed under vacuum at 30 mbar for 1 h, and deionized water was then added to cover their upper surface (water layer of 2 cm). The mortars were left for 2 more hours in the vacuum chamber to let water saturate the open porosity; then, the supernatant was removed, and the samples were placed again in the reactors connected to GC. The gas measurement protocol described in Section 2.3 was applied for a new period of 4 months. The H_2_ release measured by GC is shown in Figure 15.

No restart of gas release occurs after water resaturation of the samples containing pure Al and Al/Mg alloys. Thus, metal passivation seems to be the primary cause of the corrosion stops. This result is consistent with the short-term observations of Perona et al. [30], who measured a decrease in the corrosion current with time for their samples cured under demineralized water.

Inversely, a small and continuous increase in the H_2_ release is observed after the resaturation of MKP mortar containing pure magnesium, meaning in this case that the corrosion slow-down observed previously was due, at least in part, to water depletion.

## 5. Conclusions

The aim of this work was to investigate the corrosion of Al/Mg alloys in MKP mortars, identified as a promising conditioning matrix for radioactive waste containing aluminum metal. Electrochemical impedance spectroscopy and gas chromatography were used to determine the evolution of the corrosion rate with ongoing reaction and its dependence on the Mg content in the alloy. The main conclusions can be summarized as follows.

- Despite the strong corrosion of Mg metal at earlier ages in MKP mortar, the electrochemical behavior of Al/Mg alloys in this matrix remains close to that of pure Al. Up to 4.5 wt%, the Mg content in Al/Mg alloys has no significant influence on the cumulative volume of dihydrogen released over 1 y.
- The corrosion rate of Al and Al/Mg alloys containing up to 4.5% Mg is reduced by ~3 orders of magnitude in hardened MKP mortar, as compared to Portland-cement-based materials. It decreases as the cement reaction progresses. The decrease is rapid during the first month and then slows down, but small evolutions are still noticed after 1 y.
- On the contrary, pure magnesium is highly corroded in the MKP matrix. Its OCP always remains below the reduction potential of water. Nevertheless, the H_2_ release tends to slow down with time. Consumption of water via the cement reaction and precipitation of a passivation layer, possibly K-struvite, likely limit the corrosion process.
- The H_2_ production over a 1 year period, calculated from the corrosion current measured using EIS, is in rather good agreement with direct gas measurements using GC, which supports the postulated corrosion mechanism and the fitting method of EIS data.
- Regarding Al/Mg alloys, the gas release measured experimentally is, however, significantly smaller than that calculated from the corrosion current, assuming oxidation of Al and Mg via water. This suggests the occurrence of another oxidizing process without any formation of H_2_, which is not simply related to the magnesium content in the alloy. Further study would be required to investigate the influence of impurities and the microstructure of Al/Mg alloys on their corrosion mechanism.

## Figures and Tables

**Table 1:** Characteristics of pure metals and Al/Mg alloys investigated in this work.

Metal		Pure Al	Al/Mg2	Al/Mg3	Al/Mg4	Al/Mg4.5	Pure Mg
Reference following ANSI H35.1			5251	5754	5086	5083	
Thickness (mm)		0.5	1.5	1.5	1.5	1.2	1.5
Supplier		Alfa Aesar	AMGC Castellet	AMGC Castellet	Neyco	Goodfellow	Goodfellow
Composition (wt%)	Al	99.90	96.80	96.30	94.90	93.70	<0.05
	Mg	<0.05	2.10	3.10	4.00	4.80	99.90
	Fe		0.50	0.24	0.28	0.37	<0.05
	Mn		0.21	0.14	0.48	0.74	
	Si		0.22	0.09	0.09	0.16	
	Cr		<0.05	0.07	0.07	0.12	
	Cu, Zn, Ca, Bi	<0.05					

**Table 2:** Mix design of MKP mortar (for ~1 L batch).

Raw Constituents	Weight (g)
MgO	129
KH_2_PO_4_	437
water	289
fly ash	567
sand	567
B(OH)_3_	11.3

## Data Availability

The data presented in this study are available on request from the corresponding author.

## Appendix A

Expression of the normalized H_2_ release measured using GC is given by:

$$V_{H_{2}/S}(t)=\frac{V_{M}}{S}\times \left(n_{H_{2}reactor}(t)+\sum_{i=0}^{n}n_{H_{2}line}(t_{i})\right)$$

with $n_{H_{2}reactor}\left(t\right)=\frac{V_{reactor}\times P_{reactor}\left(t\right)\times [H_{2}](t)}{R\times T(t)}$ et $n_{H_{2}line}\left(t_{i}\right)=\frac{V_{line}\times P_{reactor}\left(t_{i}\right)\times [H_{2}](t_{i})}{R\times T(t_{i})}$, and $V_{M}$ the molar volume of H_2_ at 1 bar and 25 °C, S the metallic surface area, $V_{reactor}$ the free volume in the GC reactor, $V_{line}$ the volume of the GC analysis line, $P_{reactor}$ the pressure, T the temperature, and $\left[H_{2}\right]$ the fraction of H_2_ in the reactor.

The 1st term $n_{H_{2}reactor}(t)$ represents the molar quantity of H_2_ in the reactor; the 2nd term represents the sum of the quantity of H_2_ in the GC injection line at each sampling time $t_{i}$, which is lost when performing the analysis.

The expression of the experimental error is:

$$\frac{∆V_{H_{2}/S}}{V_{H_{2}/S}}=\frac{∆S}{S}+\frac{∆n_{H_{2}reactor}(t)}{n_{H_{2}reactor}\left(t\right)+\sum_{i=0}^{n}n_{H_{2}line}\left(t_{i}\right)}+\frac{\sum_{i=0}^{n}{∆n}_{H_{2}line}\left(t_{i}\right)}{n_{H_{2}reactor}\left(t\right)+\sum_{i=0}^{n}n_{H_{2}line}\left(t_{i}\right)}$$

with:

$$∆n_{H_{2}reactor}\left(t\right)=n_{H_{2}reactor}\left(t\right)\times \left(\frac{{∆P}_{reactor}\left(t\right)}{P_{reactor}\left(t\right)}+\frac{∆\left[H_{2}\right]\left(t\right)}{\left[H_{2}\right]\left(t\right)}+\frac{∆V_{reactor}}{V_{reactor}}+\frac{∆T(t)}{T(t)}\right)\;\sum_{i=0}^{n}{∆n}_{H_{2}line}\left(t\right)=\sum_{i=0}^{n}n_{H_{2}line}\left(t_{i}\right)\times \sum_{i=0}^{n}\left\{\frac{{∆P}_{reactor}\left(t_{i}\right)}{P_{reactor}\left(t_{i}\right)}+\frac{∆\left[H_{2}\right]\left(t_{i}\right)}{\left[H_{2}\right]\left(t_{i}\right)}+\frac{∆V_{line}}{V_{line}}+\frac{∆T(t_{i})}{T\left(t_{i}\right)}\right\}$$

- ∆S/S=5.5%, experimental error on the metallic surface area,
- $∆P_{reactor}/P_{reactor}=0.15\%$, experimental error on the pressure in the reactor,
- $∆{[H}_{2}]/\left[H_{2}\right]=3\%$, experimental error on the H_2_ fraction in the headspace,
- $∆V_{reactor}/V_{reactor}=2.5\%$, experimental error on the volume of the headspace in the reactor,
- ∆T/T=0.07%, experimental error on the temperature,
- $∆V_{line}/V_{line}=0.9\%$, experimental error on the volume of the GC analysis line.

## Appendix C

**Table A1:** Set of electric parameters used to fit EIS experimental data for pure Al 99.996 wt% embedded in MKP mortar (surface S = 2.94 cm²).

Curing Time	OCP	$C_{m}$	$R_{e}$	$R_{p1}$	$C_{p1}$	$R_{p2}$	$C_{p2}$	$C_{dl}$	β	$R_{f}$	$∆R_{f}$
Days	V/NHE	F	Ω	Ω	F	Ω	F	$F.s^{\beta -1}$		Ω	%
1	−0.80	1.4 × 10^−10^	54	277	1.1 × 10^−6^	367	3.2 × 10^−8^	6.2 × 10^−5^	0.90	1.0 × 10^4^	5%
2	−0.67	2.3 × 10^−10^	129	562	1.0 × 10^−6^	873	3.0 × 10^−8^	5.0 × 10^−5^	0.91	4.1 × 10^4^	2%
3	−0.53	2.4 × 10^−10^	159	621	1.1 × 10^−6^	969	3.1 × 10^−8^	4.6 × 10^−5^	0.89	1.3 × 10^5^	4%
6	−0.62	2.4 × 10^−10^	197	731	1.2 × 10^−6^	1180	3.2 × 10^−8^	4.5 × 10^−5^	0.89	3.4 × 10^5^	3%
7	−0.61	2.4 × 10^−10^	196	704	1.3 × 10^−6^	1150	3.3 × 10^−8^	4.6 × 10^−5^	0.88	3.2 × 10^5^	3%
14	−0.60	2.4 × 10^−10^	282	942	1.4 × 10^−6^	1560	3.5 × 10^−8^	4.2 × 10^−5^	0.88	1.4 × 10^6^	7%
21	−0.59	2.5 × 10^−10^	313	1060	1.4 × 10^−6^	1710	4.0 × 10^−8^	4.1 × 10^−5^	0.87	3.0 × 10^6^	17%
28	−0.57	2.4 × 10^−10^	318	1060	1.5 × 10^−6^	1720	4.1 × 10^−8^	4.1 × 10^−5^	0.87	2.0 × 10^6^	15%
59	−0.49	2.4 × 10^−10^	389	1270	1.5 × 10^−6^	2070	4.0 × 10^−8^	3.9 × 10^−5^	0.86	5.0 × 10^6^	10%
91	−0.46	2.3 × 10^−10^	426	1440	1.5 × 10^−6^	2300	4.1 × 10^−8^	3.8 × 10^−5^	0.86	5.0 × 10^6^	20%
118	−0.39	2.3 × 10^−10^	485	1720	1.3 × 10^−6^	2740	3.7 × 10^−8^	3.7 × 10^−5^	0.85	5.0 × 10^6^	20%
148	−0.41	2.3 × 10^−10^	431	1600	1.3 × 10^−6^	2540	3.5 × 10^−8^	3.8 × 10^−5^	0.85	1.3 × 10^6^	8%
181	−0.37	2.2 × 10^−10^	507	2060	1.0 × 10^−6^	3260	2.9 × 10^−8^	3.5 × 10^−5^	0.84	3.0 × 10^6^	33%
211	−0.32	2.2 × 10^−10^	572	2430	9.8 × 10^−7^	3800	2.8 × 10^−8^	3.4 × 10^−5^	0.83	1.0 × 10^7^	20%
244	−0.35	2.0 × 10^−10^	555	2420	9.0 × 10^−7^	3710	2.5 × 10^−8^	3.4 × 10^−5^	0.84	1.0 × 10^7^	30%
279	−0.21	1.9 × 10^−10^	753	2740	1.2 × 10^−6^	4080	4.1 × 10^−8^	2.9 × 10^−5^	0.84	4.0 × 10^7^	25%
308	−0.30	1.9 × 10^−10^	787	3130	1.2 × 10^−6^	4660	4.1 × 10^−8^	2.7 × 10^−5^	0.85	4.0 × 10^7^	25%
338	−0.29	1.8 × 10^−10^	831	3430	1.1 × 10^−6^	5040	3.8 × 10^−8^	2.7 × 10^−5^	0.85	4.0 × 10^7^	25%

**Table A2:** Set of electric parameters used to fit EIS experimental data for Al/Mg 2 wt% embedded in MKP mortar (surface S = 3.56 cm^2^).

Curing Time	OCP	$C_{m}$	$R_{e}$	$R_{p1}$	$C_{p1}$	$R_{p2}$	$C_{p2}$	$C_{dl}$	β	$R_{f}$	$∆R_{f}$
Days	V/NHE	F	Ω	Ω	F	Ω	F	$F.s^{\beta -1}$		Ω	%
1	−0.70	1.5 × 10^−10^	35	192	1.8 × 10^−6^	259	5.9 × 10^−8^	6.3 × 10^−5^	0.89	8.1 × 10^3^	2%
2	−0.52	2.9 × 10^−10^	85	680	5.2 × 10^−7^	950	2.0 × 10^−8^	5.0 × 10^−5^	0.90	2.5 × 10^4^	4%
3	−0.31	3.0 × 10^−10^	101	762	5.0 × 10^−7^	1080	1.9 × 10^−8^	4.5 × 10^−5^	0.88	5.3 × 10^4^	2%
6	−0.22	3.1 × 10^−10^	123	868	5.7 × 10^−7^	1300	2.1 × 10^−8^	4.0 × 10^−5^	0.88	1.3 × 10^5^	2%
7	−0.24	3.0 × 10^−10^	120	800	6.5 × 10^−7^	1240	2.2 × 10^−8^	3.9 × 10^−5^	0.88	1.3 × 10^5^	4%
14	−0.16	3.1 × 10^−10^	170	1100	7.0 × 10^−7^	1700	2.2 × 10^−8^	3.5 × 10^−5^	0.88	5.7 × 10^5^	4%
21	−0.16	3.1 × 10^−10^	179	1050	1.0 × 10^−6^	1600	3.0 × 10^−8^	3.4 × 10^−5^	0.88	7.0 × 10^5^	7%
28	−0.16	3.1 × 10^−10^	190	1210	7.5 × 10^−7^	1910	2.2 × 10^−8^	3.2 × 10^−5^	0.88	1.1 × 10^6^	9%
59	−0.12	3.0 × 10^−10^	240	1520	7.8 × 10^−7^	2330	2.3 × 10^−8^	3.0 × 10^−5^	0.88	2.7 × 10^6^	11%
91	−0.10	3.0 × 10^−10^	267	1780	8.0 × 10^−7^	2580	2.8 × 10^−8^	2.8 × 10^−5^	0.88	3.0 × 10^6^	17%
118	−0.03	2.9 × 10^−10^	333	2180	9.0 × 10^−7^	3250	3.1 × 10^−8^	2.6 × 10^−5^	0.88	5.0 × 10^7^	40%
148	−0.06	2.9 × 10^−10^	299	2090	7.8 × 10^−7^	2950	2.9 × 10^−8^	2.7 × 10^−5^	0.88	5.0 × 10^6^	40%
181	0.04	2.8 × 10^−10^	381	3870	6.9 × 10^−7^	4080	2.7 × 10^−8^	2.5 × 10^−5^	0.88	8.0 × 10^6^	25%
211	0.12	2.7 × 10^−10^	474	3440	7.7 × 10^−7^	5030	2.8 × 10^−8^	2.3 × 10^−5^	0.88	2.0 × 10^7^	25%
244	0.06	2.8 × 10^−10^	476	3870	4.0 × 10^−7^	5390	1.4 × 10^−8^	2.2 × 10^−5^	0.89	1.1 × 10^7^	27%
279	0.16	2.9 × 10^−10^	663	5090	6.3 × 10^−7^	7020	3.1 × 10^−8^	2.1 × 10^−5^	0.89	2.0 × 10^7^	25%
308	0.16	2.8 × 10^−10^	750	5680	6.7 × 10^−7^	7830	3.2 × 10^−8^	2.1 × 10^−5^	0.89	3.0 × 10^7^	33%
338	0.18	2.7 × 10^−10^	905	7190	5.9 × 10^−7^	10,000	2.9 × 10^−8^	2.0 × 10^−5^	0.89	5.0 × 10^7^	40%

**Table A3:** Set of electric parameters used to fit EIS experimental data for Al/Mg 3 wt% embedded in MKP mortar (surface S = 3.48 cm^2^).

Curing Time	OCP	$C_{m}$	$R_{e}$	$R_{p1}$	$C_{p1}$	$R_{p2}$	$C_{p2}$	$C_{dl}$	β	$R_{f}$	$∆R_{f}$
Days	V/NHE	F	Ω	Ω	F	Ω	F	$F.s^{\beta -1}$		Ω	%
1	−0.73	1.4 × 10^−10^	52	276	1.3 × 10^−6^	402	4.1 × 10^−8^	5.3 × 10^−5^	0.92	6.0 × 10^3^	2%
2	−0.65	2.1 × 10^−10^	110	825	6.6 × 10^−7^	1280	1.5 × 10^−8^	4.5 × 10^−5^	0.92	2.5 × 10^4^	2%
3	−0.58	2.1 × 10^−10^	126	1100	2.8 × 10^−7^	1890	6.5 × 10^−9^	4.1 × 10^−5^	0.91	5.3 × 10^4^	2%
6	−0.49	2.1 × 10^−10^	155	1300	3.5 × 10^−7^	2060	8.9 × 10^−9^	3.8 × 10^−5^	0.90	9.7 × 10^4^	2%
7	−0.53	2.1 × 10^−10^	150	1260	3.6 × 10^−7^	1950	9.5 × 10^−9^	3.7 × 10^−5^	0.90	8.5 × 10^4^	2%
14	−0.52	2.2 × 10^−10^	217	1920	3.2 × 10^−7^	3070	7.5 × 10^−9^	3.5 × 10^−5^	0.89	2.7 × 10^5^	4%
21	−0.54	2.1 × 10^−10^	228	1980	3.3 × 10^−7^	3240	8.0 × 10^−9^	3.5 × 10^−5^	0.89	2.7 × 10^5^	7%
28	−0.55	2.1 × 10^−10^	248	2210	3.0 × 10^−7^	3650	7.1 × 10^−9^	3.4 × 10^−5^	0.89	3.1 × 10^5^	5%
59	−0.35	2.0 × 10^−10^	317	3330	2.3 × 10^−7^	5150	6.3 × 10^−9^	3.4 × 10^−5^	0.86	4.1 × 10^5^	2%
91	−0.45	1.9 × 10^−10^	376	3500	3.0 × 10^−7^	5740	7.3 × 10^−9^	2.8 × 10^−5^	0.89	6.1 × 10^5^	3%
118	−0.34	1.9 × 10^−10^	466	4430	2.8 × 10^−7^	7310	7.5 × 10^−9^	2.6 × 10^−5^	0.88	8.0 × 10^5^	6%
148	−0.35	1.8 × 10^−10^	440	4360	2.5 × 10^−7^	6780	7.8 × 10^−9^	2.7 × 10^−5^	0.89	7.0 × 10^5^	7%
181	−0.18	1.8 × 10^−10^	585	5620	2.6 × 10^−7^	9220	7.5 × 10^−9^	2.2 × 10^−5^	0.90	1.4 × 10^6^	7%
211	−0.06	1.7 × 10^−10^	747	6990	2.7 × 10^−7^	11,800	8.5 × 10^−9^	1.9 × 10^−5^	0.91	2.6 × 10^6^	12%
244	−0.10	1.7 × 10^−10^	798	7840	2.5 × 10^−7^	10,500	1.1 × 10^−8^	1.9 × 10^−5^	0.91	2.8 × 10^6^	11%
279	−0.02	1.7 × 10^−10^	1090	10,,200	2.8 × 10^−7^	14,600	1.2 × 10^−8^	1.7 × 10^−5^	0.91	5.0 × 10^6^	20%
308	−0.01	1.6 × 10^−10^	1180	11,200	2.8 × 10^−7^	15,800	1.2 × 10^−8^	1.7 × 10^−5^	0.91	6.0 × 10^6^	17%
338	0.01	1.6 × 10^−10^	1410	14,300	2.5 × 10^−7^	20,600	1.2 × 10^−8^	1.7 × 10^−5^	0.91	9.0 × 10^6^	22%

**Table A4:** Set of electric parameters used to fit EIS experimental data for Al/Mg 4 wt% embedded in MKP mortar (surface S = 3.65 cm^2^).

Curing Time	OCP	$C_{m}$	$R_{e}$	$R_{p1}$	$C_{p1}$	$R_{p2}$	$C_{p2}$	$C_{dl}$	β	$R_{f}$	$∆R_{f}$
Days	V/NHE	F	Ω	Ω	F	Ω	F	$F.s^{\beta -1}$		Ω	%
1	−0.72	2.3 × 10^−10^	55	427	7.0 × 10^−7^	551	2.5 × 10^−8^	5.9 × 10^−5^	0.91	5.7 × 10^3^	2%
2	−0.49	2.5 × 10^−10^	100	673	7.8 × 10^−7^	1080	1.9 × 10^−8^	4.7 × 10^−5^	0.90	3.0 × 10^4^	3%
3	−0.44	2.6 × 10^−10^	113	921	4.1 × 10^−7^	1450	1.4 × 10^−8^	4.2 × 10^−5^	0.89	5.0 × 10^4^	2%
6	−0.41	2.6 × 10^−10^	140	1070	4.8 × 10^−7^	1720	1.7 × 10^−8^	3.8 × 10^−5^	0.89	9.8 × 10^4^	2%
7	−0.44	2.6 × 10^−10^	136	1030	5.2 × 10^−7^	1600	2.0 × 10^−8^	3.8 × 10^−5^	0.89	1.0 × 10^5^	2%
14	−0.31	2.6 × 10^−10^	195	1390	5.0 × 10^−7^	2310	1.4 × 10^−8^	3.4 × 10^−5^	0.88	2.5 × 10^5^	2%
21	−0.45	2.6 × 10^−10^	206	1440	5.0 × 10^−7^	2530	1.3 × 10^−8^	3.3 × 10^−5^	0.89	3.6 × 10^5^	3%
28	−0.46	2.5 × 10^−10^	225	1560	5.2 × 10^−7^	2690	1.4 × 10^−8^	3.2 × 10^−5^	0.89	4.6 × 10^5^	4%
59	−0.39	2.5 × 10^−10^	280	1930	5.1 × 10^−7^	3230	1.3 × 10^−8^	3.1 × 10^−5^	0.88	6.8 × 10^5^	4%
91	−0.33	2.5 × 10^−10^	309	2170	5.0 × 10^−7^	3500	1.6 × 10^−8^	2.9 × 10^−5^	0.88	9.0 × 10^5^	6%
118	−0.26	2.4 × 10^−10^	373	2620	4.7 × 10^−7^	4320	1.5 × 10^−8^	2.6 × 10^−5^	0.88	1.3 × 10^6^	8%
148	−0.27	2.4 × 10^−10^	344	2360	5.1 × 10^−7^	3850	1.6 × 10^−8^	2.7 × 10^−5^	0.89	1.1 × 10^6^	5%
181	−0.15	2.3 × 10^−10^	424	2850	4.7 × 10^−7^	4740	1.4 × 10^−8^	2.4 × 10^−5^	0.89	1.4 × 10^6^	7%
211	−0.05	2.3 × 10^−10^	514	3290	5.1 × 10^−7^	5520	1.7 × 10^−8^	2.1 × 10^−5^	0.90	1.9 × 10^6^	11%
244	−0.07	2.4 × 10^−10^	516	3250	4.3 × 10^−7^	5260	1.1 × 10^−8^	2.0 × 10^−5^	0.90	1.8 × 10^6^	11%
279	−0.01	2.3 × 10^−10^	628	3910	5.4 × 10^−7^	6010	2.2 × 10^−8^	1.9 × 10^−5^	0.91	2.3 × 10^6^	9%
308	0.08	2.3 × 10^−10^	850	5040	5.7 × 10^−7^	8160	2.2 × 10^−8^	1.8 × 10^−5^	0.91	4.0 × 10^6^	13%
338	0.00	2.3 × 10^−10^	787	4950	5.0 × 10^−7^	7780	2.0 × 10^−8^	1.8 × 10^−5^	0.91	3.0 × 10^6^	17%

**Table A5:** Set of electric parameters used to fit EIS experimental data for Al/Mg 4.5 wt% embedded in MKP mortar (surface S = 3.48 cm²).

Curing Time	OCP	$C_{m}$	$R_{e}$	$R_{p1}$	$C_{p1}$	$R_{p2}$	$C_{p2}$	$C_{dl}$	β	$R_{f}$	$∆R_{f}$
Days	V/NHE	F	Ω	Ω	F	Ω	F	$F.s^{\beta -1}$		Ω	%
1	−0.69	2.6 × 10^−10^	51	422	7.7 × 10^−7^	488	3.4 × 10^−8^	4.5 × 10^−5^	0.91	8.0 × 10^3^	3%
2	−0.52	3.0 × 10^−10^	88	497	1.2 × 10^−6^	737	4.0 × 10^−8^	3.6 × 10^−5^	0.91	3.6 × 10^4^	3%
3	−0.46	3.0 × 10^−10^	101	620	8.6 × 10^−7^	901	3.0 × 10^−8^	3.3 × 10^−5^	0.90	6.4 × 10^4^	3%
6	−0.42	3.1 × 10^−10^	120	816	5.2 × 10^−7^	1270	2.1 × 10^−8^	3.0 × 10^−5^	0.90	1.5 × 10^5^	3%
7	−0.45	3.1 × 10^−10^	116	770	5.7 × 10^−7^	1230	2.3 × 10^−8^	3.0 × 10^−5^	0.90	1.5 × 10^5^	2%
14	−0.44	3.2 × 10^−10^	169	1060	5.5 × 10^−7^	1680	2.5 × 10^−8^	2.6 × 10^−5^	0.90	5.2 × 10^5^	2%
21	−0.47	3.2 × 10^−10^	200	1200	5.5 × 10^−7^	1810	1.4 × 10^−8^	2.5 × 10^−5^	0.90	8.5 × 10^5^	2%
28	−0.52	3.2 × 10^−10^	200	1270	7.0 × 10^−7^	2040	2.5 × 10^−8^	2.6 × 10^−5^	0.90	6.2 × 10^5^	3%
59	−0.47	3.1 × 10^−10^	256	1640	7.3 × 10^−7^	2590	2.6 × 10^−8^	2.5 × 10^−5^	0.90	8.0 × 10^5^	6%
91	−0.41	3.1 × 10^−10^	292	1920	7.7 × 10^−7^	2950	3.0 × 10^−8^	2.4 × 10^−5^	0.89	8.3 × 10^5^	6%
118	−0.28	3.1 × 10^−10^	362	2600	5.6 × 10^−7^	4060	2.3 × 10^−8^	2.2 × 10^−5^	0.89	1.3 × 10^6^	8%
148	−0.31	3.0 × 10^−10^	364	2300	7.8 × 10^−7^	3670	3.2 × 10^−8^	2.2 × 10^−5^	0.90	1.2 × 10^6^	8%
181	−0.13	3.0 × 10^−10^	519	2810	8.6 × 10^−7^	4990	3.3 × 10^−8^	1.8 × 10^−5^	0.90	1.9 × 10^6^	11%
211	−0.04	2.9 × 10^−10^	678	3450	9.4 × 10^−7^	6310	3.8 × 10^−8^	1.6 × 10^−5^	0.91	3.7 × 10^6^	11%
244	−0.05	3.0 × 10^−10^	782	3400	1.2 × 10^−6^	6430	4.3 × 10^−8^	1.6 × 10^−5^	0.91	4.0 × 10^6^	13%
279	0.07	3.0 × 10^−10^	1150	4170	1.5 × 10^−6^	8850	5.6 × 10^−8^	1.5 × 10^−5^	0.91	1.3 × 10^7^	15%
308	0.09	2.8 × 10^−10^	1620	4970	2.0 × 10^−6^	11,300	7.2 × 10^−8^	1.4 × 10^−5^	0.91	4.0 × 10^7^	25%
338	0.02	2.8 × 10^−10^	1320	5260	1.3 × 10^−6^	10,900	4.8 × 10^−8^	1.4 × 10^−5^	0.91	1.5 × 10^7^	33%
